# Assessing Effects of Riboflavin/UV‐A Cross‐Linking on Aqueous Outflow Facility, Corneal Biomechanics and Their Association With Intraocular Pressure

**DOI:** 10.1002/jbio.202500432

**Published:** 2025-10-29

**Authors:** Yilong Zhang, Zhengshuyi Feng, Xingyu Jiang, Robert Scott, Ying Yang, Chunhui Li, Zhihong Huang

**Affiliations:** ^1^ School of Physics, Engineering and Technology University of York York UK; ^2^ Centre for Medical Engineering and Technology, School of Science and Engineering University of Dundee Dundee UK; ^3^ Théa Pharmaceuticals Ltd Keele UK; ^4^ School of Pharmacy and Bioengineering Keele University Stoke‐on‐Trent UK

**Keywords:** air‐pulse optical coherence elastography, biomechanical properties, intraocular pressure, keratoconus, manometric measurement, outflow facility, riboflavin/UV‐A corneal collagen cross‐linking

## Abstract

Riboflavin/UV‐A corneal collagen cross‐linking (CXL) is a standard treatment for early‐stage keratoconus. However, quantitative CXL outcomes remain limited. This study evaluated CXL effects on outflow facility and corneal biomechanics at intraocular pressures (IOPs) in ex vivo porcine eyes. Ocular rigidity and outflow facility were derived from pressure–volume and IOP decay curves using a direct manometric technique, while corneal elasticity (Young's modulus) was measured via non‐contact air‐pulse optical coherence elastography. CXL increased ocular rigidity (0.0066 ± 0.0001 μL^−1^ vs. 0.0060 ± 0.0002 μL^−1^) and reduced outflow facility from 0.5429 ± 0.0320 to 0.1485 ± 0.0153 μL/min/mmHg (20–40 mmHg), compared to 0.7327 ± 0.0894–0.2210 ± 0.0502 μL/min/mmHg in untreated eyes. Young's modulus increased by 92%, 89%, and 155% at 20, 30, and 40 mmHg. These findings enhance our understanding of flow dynamics at IOP levels, suggesting that outflow facility and corneal biomechanics may serve as potential indicators for evaluating the effectiveness of CXL.

## Graphical abstract

Our study demonstrates that UV cross‐linking (CXL) reduces outflow facility while increasing corneal elasticity across IOP levels of 20–40 mmHg, measured using direct manometry and non‐contact optical coherence elastography. These results highlight both parameters as potential indicators for evaluating CXL effectiveness and monitoring keratoconus treatment outcomes.

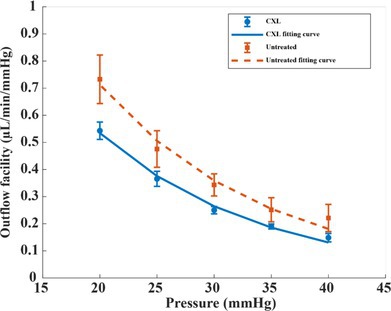

## 1 Introduction

Keratoconus is a bilateral, degenerative corneal disorder characterized by localized corneal thinning and a conical protrusion of the cornea, leading to reduced visual acuity due to irregular astigmatism and high myopia [1]. Although it was once considered a non‐inflammatory condition, this view is now challenged by the identification of proinflammatory mediators involved in its pathogenesis [2, 3]. It is the most prevalent form of corneal ectasia and affects individuals of all ethnicities [4]. Riboflavin/UV‐A corneal collagen cross‐linking (CXL) has become a standard clinical procedure to enhance the biomechanical stability and rigidity of the cornea, thereby stopping or slowing disease progression in keratoconus and preventing further deterioration [5]. The procedure induces cross‐links between collagen fibrils and within the proteoglycan‐rich matrix, as well as among collagen molecules and proteoglycan core proteins [6]. However, there are limited quantitative parameters available to evaluate the outcomes of CXL interventions in keratoconus, and conflicting results have been reported depending on the device used to assess corneal biomechanics. While several studies using the Ocular Response Analyzer (ORA) have shown that corneal hysteresis does not appear to change significantly after CXL [7, 8, 9], other studies using the Corvis ST have reported changes in several biomechanical parameters [9, 10, 11].

Research has shown that CXL influences aqueous outflow resistance [12]. Aqueous outflow occurs through the trabecular meshwork (the so‐called ‘conventional pathway’) and through the uveoscleral route (the so‐called ‘unconventional pathway’) [13]. Impaired aqueous humor outflow decreases outflow facility and elevates intraocular pressure (IOP) [14]. Consequently, measuring outflow facility is clinically important for identifying glaucoma risk [15]. Other ocular conditions, such as nonproliferative diabetic retinopathy [16] and uveitis [17], also affect outflow facility.

Outflow facility reflects the eye's ability to drain aqueous humor and is quantified by the relationship between the outflow rate of aqueous humor and the intraocular pressure (IOP). A higher facility value corresponds to lower resistance within the drainage pathways, enabling more efficient aqueous humor removal and contributing to stable IOP regulation [18]. It is normally measured using tonography, where sustained pressure is applied to the eye and the resulting IOP and volume changes are recorded [19]. Outflow facility can also be measured by a direct tonographic technique via inserting a manometric device into the anterior chamber, which offers direct and precise measurements of both IOP and ocular rigidity [20, 21].

There has been increased interest in assessing corneal biomechanics for detecting keratoconus [22] and evaluating CXL treatment [23]. Building on the established success of optical coherence tomography (OCT) in ophthalmology, optical coherence elastography (OCE), with its potential for high resolution and high sensitivity, has been recognized for its promise in clinical corneal biomechanical assessment [24, 25]. Air‐pulse OCE, in particular, has demonstrated accurate biomechanical characterization of CXL treatment in corneas [26], using a safe and low‐pressure micro air‐pulse delivery method [27].

In this study, we present the first study on the effects of CXL on outflow facility in ex vivo porcine eyes, and their dependence on IOP, using a direct manometric technique. We further use a non‐contact air‐pulse OCE approach to quantify corneal biomechanical changes in response to CXL treatment and IOP elevations, to explore the correlation with outflow facility. This study highlights outflow facility and corneal biomechanical properties as potential indicators for evaluating the effectiveness of CXL treatment and monitoring responses in keratoconus management.

## 2 Materials and Methods

### 2.1 Sample Preparation

Twenty fresh porcine globes were obtained from a local supplier (Medmeat Ltd., UK) and tested within 24 h of enucleation. All external tissues (fat and muscle) were manually removed, and the intact globes were placed in an eyeball stand (I‐STAND, Madhu Instruments Pvt. Ltd., India) for both manometric and OCE experiments.

### 2.2 Riboflavin/UV‐A Corneal Collagen Cross‐Linking

CXL was performed by a technique that mimics the standard “Dresden” protocol [28]. First, the corneal epithelium was removed with a blunt surgical instrument. A 0.1% riboflavin solution in 20% dextran was then applied topically to the cornea every 5 min for 30 min. The cornea was subsequently irradiated at 365 nm (2.74 mW/cm^2^) for 33 min, with additional riboflavin instilled every 5 min during the irradiation. All measurements were performed immediately after the CXL procedure was completed.

### 2.3 Manometric Device and Measurement Procedure

**FIGURE 1:**
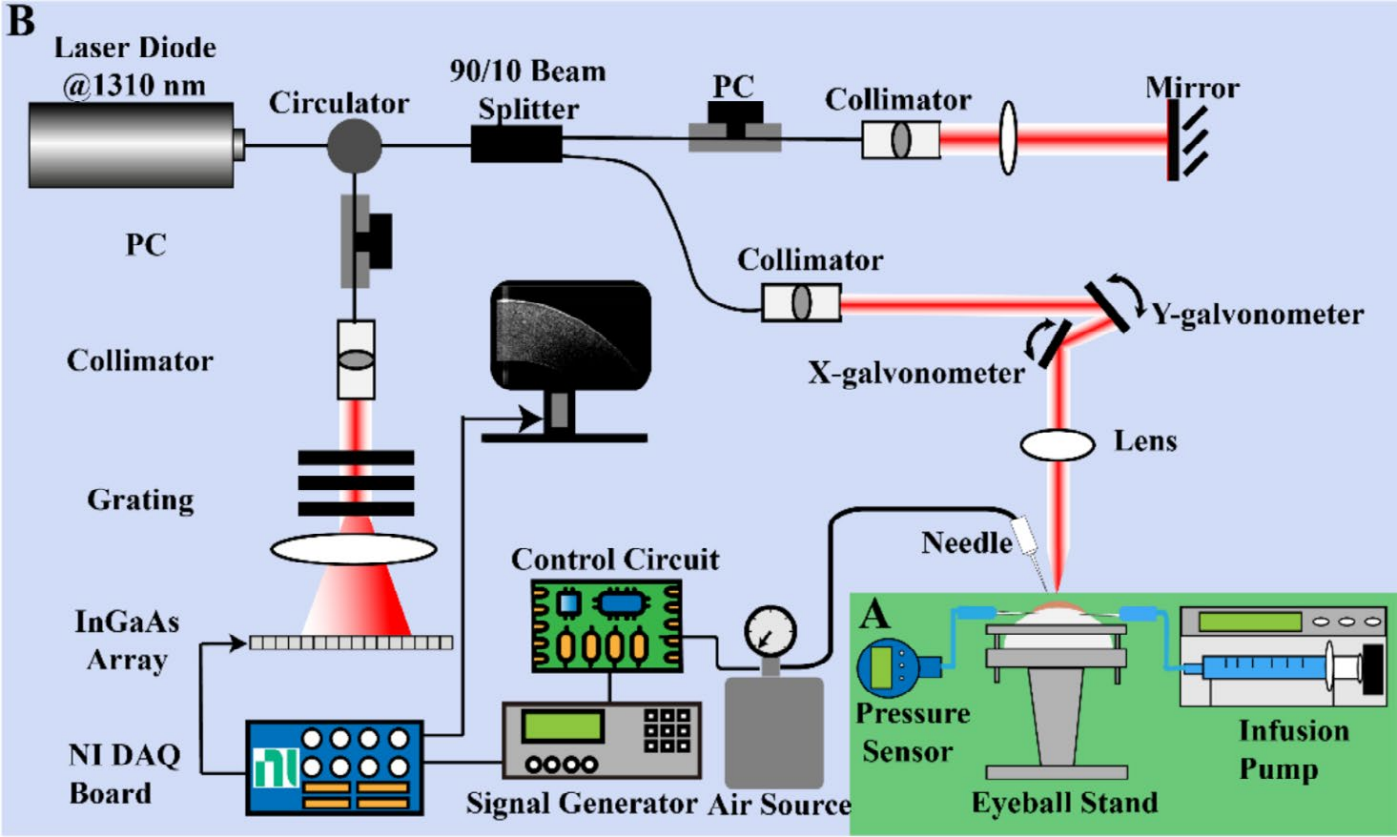
Schematic of the experimental setup of manometric devices (A: Green) and non‐contact OCE system (B: Blue). The air‐pulse OCE system consisting of a PhS‐OCT system and an air‐pulse stimulation system. DAQ, data acquisition; NI, national instrument; PC: polarization controller; PhS‐OCT, phase‐sensitive optical coherence tomography; SAW‐OCE, surface acoustic wave optical coherence elastography.

All eyes were measured in situ using manometric devices (Figure 1A) that simulated and recorded IOP in each sample. A micro‐infusion pump (Fresenius Kabi AG, USA) was connected via tubing to a 23G needle, which was inserted into the anterior chamber of the porcine eye. The infusion pump delivered 1× Phosphate‐Buffered Saline (PBS) (Sigma‐Aldrich, Dorset, UK) to adjust the IOP. Another 23G needle was connected via tubing to a digital pressure gauge (Keller LeoRecord, BSRIA Limited, Bracknell, UK) to measure the intraocular pressure. The readings of the pressure gauge were recorded by a corresponding interface, PressureSuite software, with a sampling rate of 14 Hz.

Eyes that underwent manometric measurements were divided into two groups: the untreated control group (*n* = 5) and the CXL treatment group (*n* = 3). The pressure was referenced (set to zero) at the point of entrance before the insertion into the anterior chamber to eliminate possible hydrostatic pressure differences between the measured eye and the pressure gauge. IOP was artificially increased to 40 mmHg by infusion steps of a known volume of saline solution in the anterior chamber of the eye, at a rate of 20 mL/h. The infusion time required to raise IOP to 40 mmHg was approximately 97 ± 8 s for untreated eyes and 122 ± 4 s for CXL‐treated eyes. This corresponded to an average infused volume of 543 ± 48 μL in normal eyes and 680 ± 24 μL in CXL‐treated eyes. When IOP reached 40 mmHg, the infusion stopped, and the IOP decay curve over time was recorded for time intervals between 3 and 4 min. The average time for the pressure to decay to 20 mmHg was 182 ± 19 s in normal eyes and 242 ± 3 s in CXL‐treated eyes. The eyes were continuously hydrated by dropping 1× PBS every 10 s.

### 2.4 Non‐Contact Air‐Pulse OCE System and Measurement Procedure

A lab‐built air‐pulse OCE system (Figure 1B) consisted of a phase‐sensitive spectral‐domain optical coherence tomography (PhS‐OCT) system and an air‐pulse stimulation system, which was described in our recent paper [29]. The theoretical lateral and axial resolutions of the system in air were ~16 μm and ~6.9 μm, respectively. The axial sampling resolution of the system was ~5.0 μm, assuming the refractive index of the whole cornea as 1.376 [30]. The air‐puff system was synchronized with the PhS‐OCT system. The outlet of the air stream was delivered from a flat edge end needle port (Weller KDS2312P, Rapid Electronics Limited, Essex, UK) with an internal diameter of 0.4 mm.

Eyes that underwent air‐pulse OCE measurements were divided into two groups: the untreated control group (*n* = 8) and the CXL treatment group (*n* = 4). Using the artificial IOP control system, measurements were performed at 20, 30, and 40 mmHg in each eye. These IOP levels were chosen to reflect clinically relevant thresholds used in glaucoma management. In humans, an IOP of 20 mmHg is regarded as at the upper level of normal, and values above this threshold would be considered to have ocular hypertension and at risk of requiring treatment for mild‐to‐moderate glaucoma [31]. IOPs above 30 mmHg are generally considered a clear indication for treatment, while pressures exceeding 40 mmHg are associated with a high risk of rapid optic nerve damage and require urgent therapeutic intervention [32]. The air‐pulse system was driven by an externally triggered function generator (33220A, Keysight Technologies Inc., California). The driving signal was a square wave with a 60 Hz frequency, with a peak‐to‐peak voltage amplitude (*Vpp*) of 5 V, the offset value (*Vdc*) is +2.5 V and the duty cycle of the wave was 50%. The excitation angle of incidence relative to the surface normal of the cornea was set to 45°. The distance between the needle tip and corneal apex was set to 0.5 mm. An M‐B scanning protocol was employed to acquire the propagation of the impulse SAW [33]. The size of the effective imaging plane was ~2.4 mm × ~9 mm (depth × lateral distance). The acquisition time for each was approximately 75.6 s. The OCE measurements were repeated at IOP increasing from 20 to 40 mmHg at increments of 10 mmHg. Hydration state is a critical factor for accurately quantifying corneal biomechanical properties [34]. To ensure that the corneas were hydrated, the corneas were dropped with 1× PBS solution every 10s and immediately after each OCE data acquisition.

### 2.5 Data Analysis

#### 2.5.1 Ocular Rigidity Coefficient Measurement

The ocular rigidity coefficient (K) was calculated via the pressure–volume relationship [35] obtained during the infusion phase. The volume change was converted according to the known infusion rate and time. The mean IOP every 2 s interval was calculated from the recorded pressure curve during infusion and plotted versus volume [21]. The experimental IOP and corresponding volume increments were then fitted using a first‐order exponential function to reduce the influence of any fluctuations in pressure readings, which was used in the manometric method for living human eyes previously [14, 20, 21], and then K can be calculated below:

(1) $$K=\frac{\mathrm{Log}P_{1}-\mathrm{Log}P_{0}}{V_{1}-V_{0}}$$

where *P*_0_ corresponds to the initial pressure of the fitting curve. *P*_1_ is the IOP at the end of the curve. *V*_1_ and *V*_0_ are the volumes in the eye when the pressure was *P*_1_ and *P*_0_.

#### 2.5.2 Outflow Facility Measurement

The outflow facility (C) was calculated at various pressures along the IOP outflow decay curve over time. According to a method proposed by Karyotakis et al. [21], the pressure data obtained during outflow measurement were smoothed using a custom exponential fit for each eye, using the following equation:

(2) $$P=P_{\mathrm{st}}+P_{i}\cdot e^{-\mathrm{at}}$$

where P is the outflow pressure at a given time, $P_{\mathrm{st}}$ is the steady‐state pressure of the eye (using the measured value of 6.3 mmHg in the enucleated porcine eyes [36]), $P_{i}$ is a pressure‐related variable, a is a time constant describing the pressure drop over time.

Then, outflow facility can be calculated from only two similar IOP values (*P*_1_, *P*_2_) from the experimental IOP decay curve as follows:

(3) $$C=\frac{\left(\frac{\mathrm{dP}}{\mathrm{dt}}\left|\;t_{1\cdot }\frac{1}{KP_{1}}-\frac{\mathrm{dP}}{\mathrm{dt}}\right|t_{2\cdot }\frac{1}{\;KP_{2}}\right)}{P_{2}-P_{1}}$$

The resulting outflow facilities from this procedure corresponded to IOP levels of 40, 35, 30, 25, and 20 mmHg for all eyes.

**FIGURE 2:**
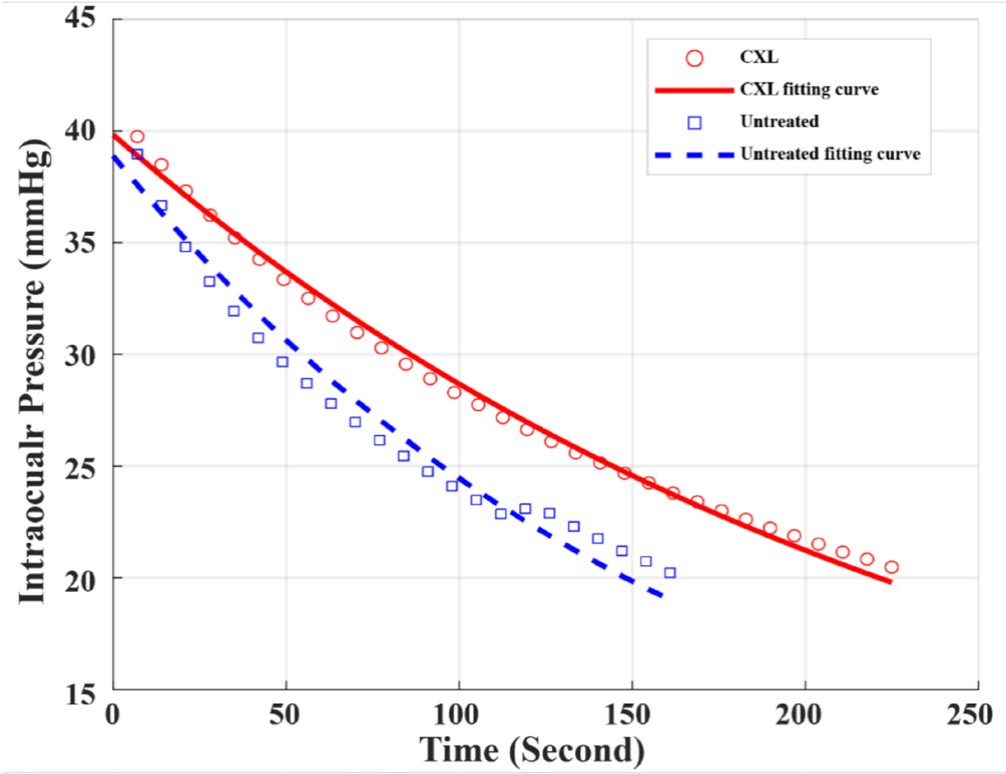
Representative IOP decay curves of untreated (blue dashed line) and CXL‐treated (red line) eyes over time.

In Figure 2, the fitting IOP decay curve of untreated eyes drops from 40 to 20 mmHg within 160 s. In comparison, the fitting IOP decay curve of CXL‐treated eyes uses approximately 230 s from 40 to 20 mmHg.

#### 2.5.3 Biomechanical Property Measurement

**FIGURE 3:**
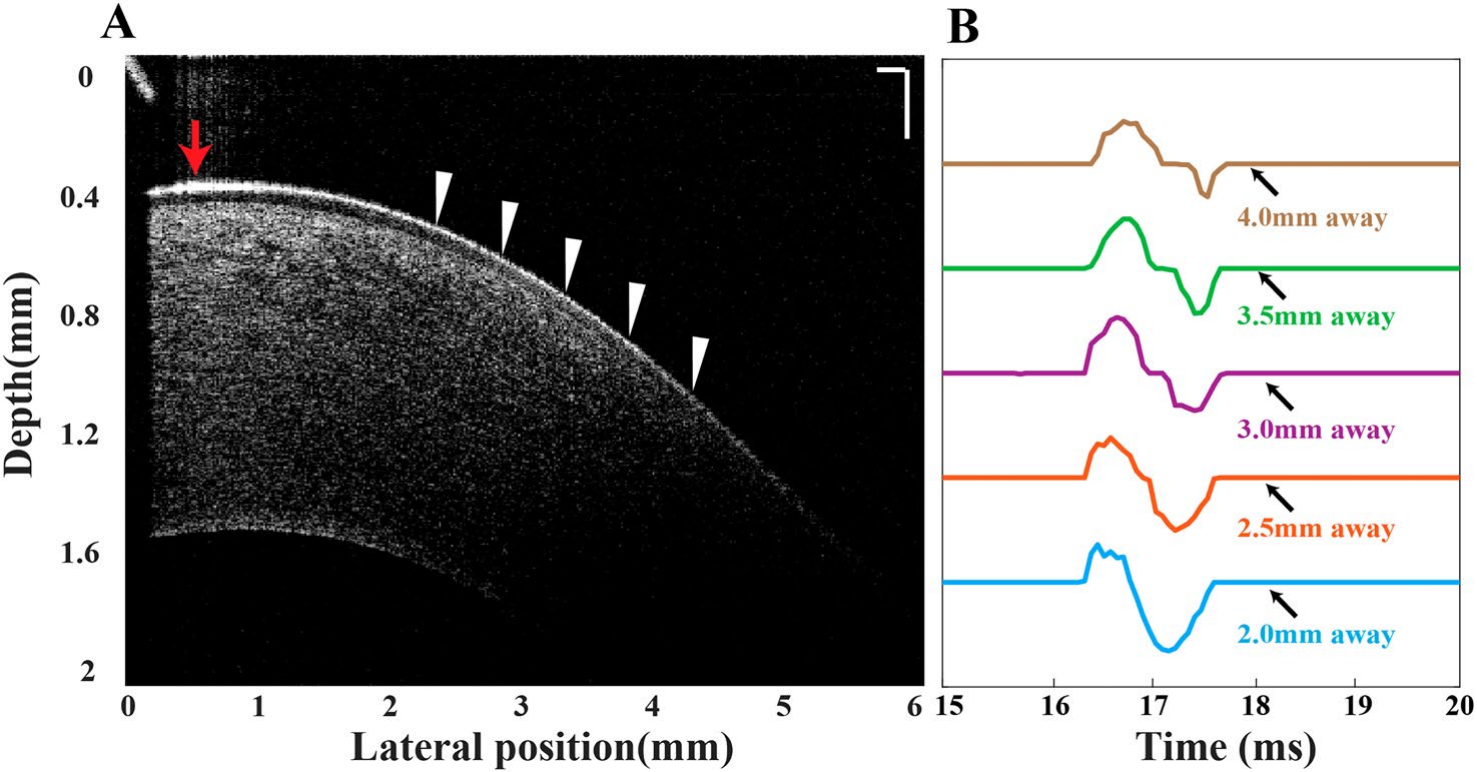
Representative OCT structural image (A) and elastic wave waveforms (B) of an untreated cornea at a given IOP at 30 mmHg. In (A), white triangles indicate measurement positions at 2, 2.5, 3.0, 3.5, and 4 mm away from the wave source (red arrow), with the corresponding waveforms shown in (B). The needle tip is visible in the upper left corner. The scale bar represents 200 μm.

Biomechanical property measurements were performed immediately after CXL, as corneal biomechanical changes occur directly following the procedure [37]. At higher frequencies, the corneal response is predominantly elastic. In this study, a purely elastic model was assumed for calculating Young's modulus, as the viscous component becomes less significant under high‐frequency excitation [38]. The elastic wave velocity (*V*_*R*_) was estimated by the slope of the space–time main wavefront peak curve along lateral locations by [39].

(4) $$V_{R}=\frac{\mathrm{\Delta x}}{\mathrm{\Delta t}}$$

where Δx is the distance traveled by the main peak of the elastic wave during time difference Δt along lateral locations, extracted from elastic wave waveforms (Figure 3).

The Young's modulus, *E*, was quantified from the elastic wave velocity by the surface acoustic wave (SAW) equation [40]:

(5) $$E=\frac{2\rho {\left(1+v\right)}^{3}}{{\left(0.87+1.12v\right)}^{2}}V_{R}^{2}$$

where *ρ* = 1062 kg/m^3^ was the density of the cornea [41] and *ν* = 0.49 was the Poisson's ratio to account for the near incompressibility of the corneal tissue [42].

The correlation between outflow facility and corneal elasticity at IOP levels of 20, 30, and 40 mmHg was assessed using pairwise correlation in MATLAB (R2023a, The MathWorks Inc., Natick, MA, USA).

## 3 Results

### 3.1 Ocular Rigidity Coefficient and Outflow Facility

Table 1 shows ocular rigidity coefficient and outflow facility of eyes untreated and treated with UV CXL. The ocular rigidity coefficient of the porcine eyes was calculated as the ratio of change in IOP to the change in volume according to Equation (1). The average OR for the CXL‐treated group was 0.0066 ± 0.0001 μL^−1^, which was higher than that of the untreated group of 0.0060 ± 0.0002 μL^−1^. The mean outflow facility coefficient for untreated eyes was 0.2210 ± 0.0502 μL/min/mmHg at 40 mmHg, increasing to a mean outflow facility coefficient of 0.7327 ± 0.0894 μL/min/mmHg at 20 mmHg. A smaller outflow facility was obtained at the given IOPs in CXL‐treated eyes. The mean outflow facility coefficient at 40 mmHg was 0.1485 ± 0.0153 μL/min/mmHg and increased to 0.1899 ± 0.0091 μL/min/mmHg, 0.2501 ± 0.0136 μL/min/mmHg, 0.3655 ± 0.0276 μL/min/mmHg, and 0.5429 ± 0.0320 μL/min/mmHg at IOPs of 35, 30, 25, and 20 mmHg, respectively.

**TABLE 1:** Ocular rigidity coefficient and outflow facility of CXL‐treated and untreated groups at given IOP levels.

IOP (mmHg)	Untreated	CXL
	Ocular rigidity (μL^−1^)	
	0.0060 ± 0.0002	0.0066 ± 0.0001
	Outflow facility (μL/min/mmHg)	
20	0.7327 ± 0.0894	0.5429 ± 0.0320
25	0.4755 ± 0.0673	0.3655 ± 0.0276
30	0.3432 ± 0.0408	0.2501 ± 0.0136
35	0.2514 ± 0.0446	0.1899 ± 0.0091
40	0.2210 ± 0.0502	0.1485 ± 0.0153

*Note:* Data are presented as mean ± standard deviation.

**FIGURE 4:**
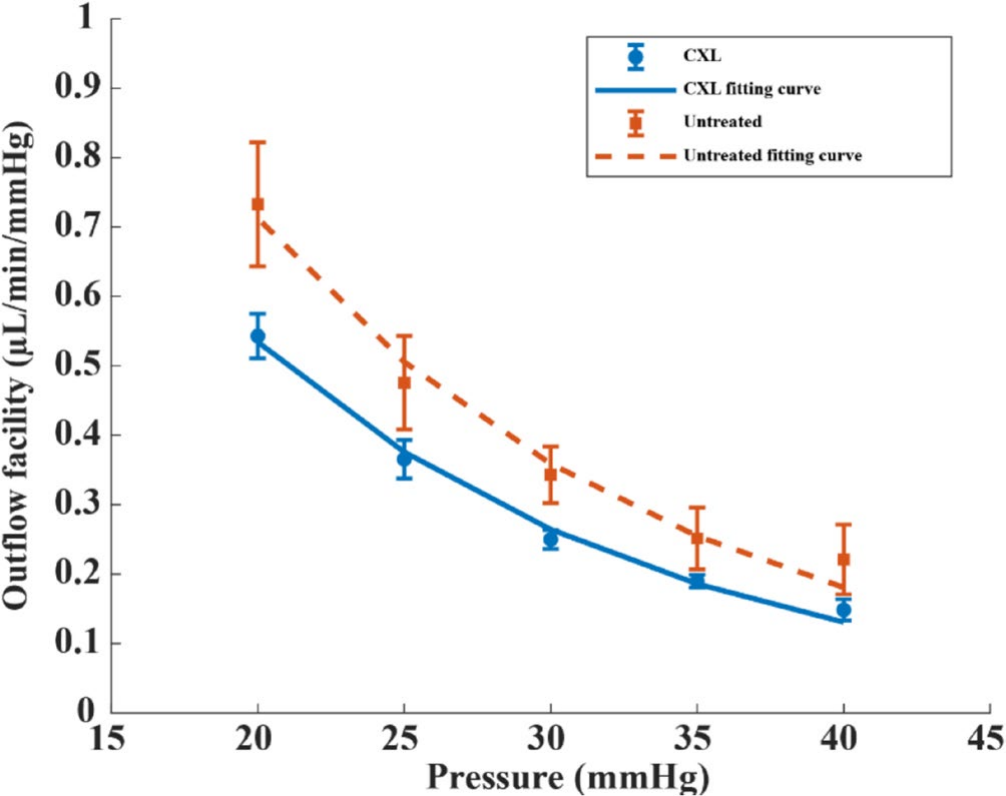
The exponential model fit of the dependence of outflow facility with increasing intraocular pressure (IOP).

Outflow facility of untreated eyes and CXL‐treated eyes was shown to have a nonlinear relationship with given IOP levels (Figure 4). An exponential mathematical formula $C=C_{0}\;e^{\left(-b\times \mathrm{IOP}\right)}$, was fitted to describe their dependency on IOP levels. The values of $C_{0}$ correspond to outflow facility at zero IOP. The coefficient of determination (*R* ^2^) was 0.97 for the untreated group and 0.99 for the CXL‐treated group. The average $C_{0}$ of untreated eyes was 2.807 μL/min/mmHg, higher than that of CXL‐treated eyes, with 2.182 μL/min/mmHg.

### 3.2 Young's Modulus of Untreated and CXL‐Treated Corneas

**FIGURE 5:**
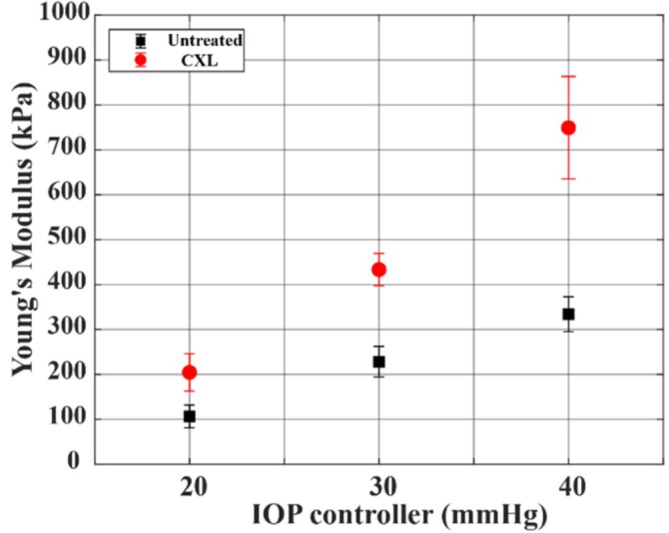
Young's modulus in untreated (black) and cross‐linking (CXL) (Red) cornea groups measured with optical coherence elastography at intraocular pressures (IOPs) from 20 to 40 mmHg, respectively.

Figure 5 shows Young's modulus of the corneas at IOPs from 20 to 40 mmHg, as quantified by the surface wave equation. The Young's moduli of the untreated corneas at 20, 30, and 40 mmHg were 106.5 ± 25.2, 228.2 ± 34.0, and 334.0 ± 38.7 kPa. After CXL, the stiffness of the corneas increased to 204.5 ± 41.6, 433.5 ± 35.9, and 749.3 ± 114.2 kPa at IOPs of 20, 30, and 40 mmHg. At IOPs of 20, 30, and 40 mmHg, the relative increase in Young's modulus was 92%, 89.9%, and 155%, respectively.

The outflow facility of untreated and CXL‐treated eyes was validated from their corneal elasticity at given IOPs of 20, 30, and 40 mmHg, through a linear dependence analysis. The untreated and CXL‐treated groups from the two measurement methods exhibited high correlation coefficients, with 97% and 93%, respectively.

## 4 Discussion

This study represents, to our knowledge, the first to measure the outflow facility of porcine eyes at evaluated IOP levels and the response to riboflavin/UV‐A CXL treatment. In addition, corneal biomechanical properties of the eyes were assessed using a non‐contact air‐pulse OCE method to validate the changes in the outflow facility. Our results demonstrated that UV‐CXL treatment would reduce the outflow facility and increase corneal biomechanical properties, which varied with IOP. Several findings emerge from this study. (1) We observed changes in the outflow facility after riboflavin/UV‐A CXL, suggesting that the CXL treatment for keratoconus patients may also influence the overall resistance to aqueous humor outflow. This finding indicates that outflow facility could be an additional parameter to evaluate CXL effectiveness and monitor treatment responses. (2) We characterized corneal Young's modulus over a range of IOPs, which could be clinically important for IOP‐related disease diagnosis. (3) We observed strong correlations between outflow facility and corneal elasticity in both untreated and CXL‐treated eyes across various IOP levels. This suggests an interplay between corneal stiffness and aqueous humor dynamics, potentially mediated by changes in the extracellular matrix. Our study demonstrates that both corneal biomechanical metrics and outflow facility have potential for the clinical evaluation of CXL effectiveness, providing support for monitoring keratoconus progression and assessing treatment response.

The ocular rigidity coefficient was calculated as the ratio of IOP change to volume change, describing the eye's overall resistance to distension. The CXL‐treated porcine eyes in our study had an ocular rigidity coefficient of 0.0066 ± 0.0001 μL^−1^, about 0.0006 μL^−1^ higher than the untreated group. These values are in the same order of magnitude as previously reported values for porcine eyes [43, 44, 45]. Regarding cross‐linking effects, Liu and He [43] observed an increase in ocular rigidity coefficient from 0.0047 ± 0.0005 μL^−1^ in untreated controls to 0.0063 ± 0.0006 μL^−1^ in globes treated with 1% glutaraldehyde. Avila et al. [45] further reported a mean ocular rigidity coefficient of 0.0065 μL^−1^ for UV‐CXL eyes, which was comparable to the value observed in our study.

Studies [21, 46, 47] have reported a decreasing trend in outflow facility with rising IOP in human eyes, consistent with our findings in porcine eyes. For example, Karyotakis et al. [21] reported that using a similar manometric method, the outflow facility coefficient in living human eyes was 0.0672 ± 0.0296 μL/min/mmHg at 40 mmHg and 0.2652 ± 0.1164 μL/min/mmHg at 20 mmHg for subjects aged 71.2 ± 4.1 years. Mammalian eyes exhibit similar outflow changes with IOP, but the outflow facility is higher than that observed in human eyes at the same pressure [48]. This difference can be attributed to anatomical variations in the aqueous outflow pathways. Porcine eyes possess a much thicker trabecular meshwork (TM) and lack a continuous Schlemm's canal [49]. These structural differences alter the resistance properties of the outflow pathway and may explain the relatively higher facility values observed in the porcine eyes in our study. Our results show a decrease in outflow facility with increasing IOP in both untreated and CXL‐treated porcine eyes. The outflow facility coefficient for untreated eyes was 0.2210 ± 0.0502 μL/min/mmHg at 40 mmHg and 0.7327 ± 0.0894 μL/min/mmHg at 20 mmHg. In CXL‐treated eyes, the outflow facility was 0.1485 ± 0.0153 μL/min/mmHg at 40 mmHg and 0.5429 ± 0.0320 μL/min/mmHg at 20 mmHg. These values are within the range of previously reported outflow facility measurements for enucleated porcine eyes, which vary between 0.29 and 1.74 μL/min/mmHg depending on test conditions [50, 51, 52, 53]. Specifically, Fujimoto et al. reported an increase in outflow facility from 0.517 ± 0.138 to 0.560 ± 0.158 μL/min/mmHg following Vascular Endothelial Growth Factor‐A treatment [50]. Strohmaier et al. measured outflow facility values of 0.31 ± 0.09 μL/min/mmHg under high flow conditions and 0.29 ± 0.03 μL/min/mmHg under low flow conditions, at baseline IOPs of 16.05 ± 3.78 and 16.56 ± 1.84 mmHg, respectively [51]. Man et al. reported mean outflow facility values of 1.74 ± 0.85 μL/min/mmHg for IOP differences between 7.86 and 10.10 mmHg [52]. Similarly, Rao et al. obtained an outflow facility of 1.09 ± 0.007 μL/min/mmHg at 15 mmHg in enucleated porcine eyes [53]. The reduction in outflow facility with increasing IOP (Figure 4) may be explained by mechanical changes in the anterior chamber angle at higher pressures, which can alter aqueous outflow pathways [54]. With respect to the changes observed after UV‐CXL, matrix cross‐linking can profoundly affect outflow resistance through modifications of ECM composition, where increased stiffness is associated with reduced outflow facility [12]. This is consistent with the paradigm that the stiffer the ECM, the lower the aqueous outflow facility [55]. Our findings extend this concept by quantifying outflow facility at different IOP levels following CXL. The results suggest that outflow facility may serve as a functional biomarker for assessing the biomechanical impact of CXL in keratoconus, offering clinically relevant information for more effective monitoring of treatment outcomes.

The biomechanical properties of untreated and CXL‐treated porcine eyes at wide IOP levels were also evaluated using the non‐contact OCE technique. Previous studies have reported Young's modulus values of approximately 70 kPa [56, 57] to 150 kPa [58] at 20 mmHg, and around 160 [56, 57] at 30 mmHg in porcine eyes. Variations in reported Young's modulus values among different studies may stem from factors such as corneal hydration [34] and storage media, age of the corneas [59] and general degradation of the ocular tissues after enucleation. Our results agreed with their values, with 106.5 ± 25.2 and 228.2 ± 34 kPa at 20 and 30 mmHg. However, corneal biomechanics at 40 mmHg have not been examined with OCE. Our value of 334.0 ± 38.7 kPa is comparable to approximately 297 kPa reported from destructive mechanical testing [60]. After CXL, the relative increase in corneal stiffness compared to untreated eyes was reported as 86% and 79% at 20 and 30 mmHg, respectively [57], which aligns closely with our observed increases of 92% and 89.9% at the same pressures. The biomechanical stiffening effect of CXL has been demonstrated not only in porcine eyes but also in human corneas. In human cadaveric corneas, studies [61, 62] have reported increases in Young's modulus of more than 300% following CXL. This increase was much greater than the ~80% increase typically observed in porcine eyes. This difference can be attributed to anatomical factors since collagen cross‐linking is most effective within the anterior 300 μm of the stroma, which represents a larger proportion of the overall thinner human cornea compared with porcine tissue [62]. This confirms that CXL will be a sensitive measure for human keratoconus cornea treatment. Recent advances in elastography, including improved noise‐removal techniques [63], could contribute to the measurements of additional biomechanical properties, such as viscoelasticity [64]. The physiological link between corneal biomechanics and outflow facility likely arises from extracellular matrix (ECM) remodeling. Alterations in matrix metalloproteinase activity and ECM protein synthesis can modify the biomechanical properties of ocular tissues and thereby influence aqueous outflow resistance [12]. This could be the explanation for the correlation coefficients of 97% and 95% between outflow facility and corneal elasticity for the untreated and CXL‐treated groups in our study.

There are several points that should be discussed or addressed in future studies. First, the relatively small sample sizes in each group restricted the statistical power for detecting significant differences between groups at specific IOP levels and for correlating outflow facility with corneal elasticity. Karyotakis et al. [21] reported a strong dependence of outflow facility on IOP levels, and in our study, we observed a similar decreasing trend in both untreated and treated groups. However, the limited sample size, especially in the CXL group, did not fully meet the requirements for robust statistical analysis. In future work, we plan to increase the number of experimental eyes to strengthen the reliability of the findings. Second, previous studies [65, 66, 67] have shown that ocular rigidity increases with IOP, and several formulas have been developed to describe this non‐linear relationship. While these formulations are more accurate, they are often complex and difficult to implement in routine experimental or clinical practice [68]. Because ocular rigidity varies considerably between individuals [69], in this study, we estimated rigidity for each eye based on the fitted pressure–volume curve in the 20–40 mmHg range, thereby reducing the bias associated with assuming a constant value. This method simplifies the rigidity measurement while preserving sufficient accuracy, making it more practical for applications. Several manometric studies [14, 21] have also adopted such simplifications for ocular rigidity estimation in outflow facility calculations. However, according to Equation (3), an underestimated rigidity at low pressures could lead to an overestimation of outflow facility, while at higher pressures, it could potentially result in an underestimation of outflow facility. Future studies should therefore aim to incorporate more precise and practical methods for capturing the true pressure dependence of ocular rigidity to improve the accuracy of outflow facility estimation. Second, in outflow facility measurements, the initial pressure and steady‐state pressure are required in the exponential function fitting process, which significantly impacts the fitting accuracy. In in vivo human studies, tonometry is commonly used before the procedure to determine these values [14, 21]. Since our study was conducted on excised porcine eyes, we adopted a general measured value of 6.3 mmHg as the initial IOP, based on previous findings from intraocular pressure measurements in enucleated porcine eyes [36]. In future studies, experiments involving living animal eye models will be essential to further validate our methodology. Third, untreated healthy porcine eyes were used as the control group in this study. While this provides results on baseline aqueous outflow and corneal biomechanics, a more comprehensive evaluation of UV‐CXL treatment effects could be achieved by incorporating porcine eyes with collagen damage as a model for pathological conditions. This would allow for a direct assessment of outflow facility and biomechanical changes before and after treatment, offering a more clinically relevant understanding of the therapeutic impact of cross‐linking.

## 5 Conclusion

In conclusion, the present study measured outflow facility and biomechanical properties in porcine eyes that underwent riboflavin/UV‐A CXL treatment, using a direct manometric method and a non‐contact optical coherence elastography technique, respectively. The results demonstrated CXL treatment reduced outflow facility compared to untreated eyes, with the effect influenced by intraocular pressure. Additionally, a strong correlation was observed between outflow facility and corneal elasticity. These findings enhance our understanding of the relationship between outflow facility and IOP in porcine eyes, providing a useful reference for research on aqueous outflow dynamics. Combining corneal biomechanical metrics with outflow facility may enable a more comprehensive clinical evaluation of CXL effectiveness, supporting monitoring of keratoconus progression, treatment response, and personalized therapeutic strategies.

## Conflicts of Interest

The authors declare no conflicts of interest.

## Data Availability

The data that support the findings of this study are available from the corresponding author upon reasonable request.
